# Role of Abl in airway hyperresponsiveness and airway remodeling

**DOI:** 10.1186/1465-9921-14-105

**Published:** 2013-10-11

**Authors:** Rachel A Cleary, Ruping Wang, Tao Wang, Dale D Tang

**Affiliations:** 1 Center for Cardiovascular Sciences, Albany Medical College, 47 New Scotland Avenue MC-8, Albany, NY 12208, USA

**Keywords:** Airway hyperresponsiveness, Airway remodeling, Tyrosine kinase, Airway smooth muscle

## Abstract

**Background:**

Asthma is a chronic disease that is characterized by airway hyperresponsiveness and airway remodeling. The underlying mechanisms that mediate the pathological processes are not fully understood. Abl is a non-receptor protein tyrosine kinase that has a role in the regulation of smooth muscle contraction and smooth muscle cell proliferation *in vitro*. The role of Abl in airway hyperresponsiveness and airway remodeling *in vivo* is largely unknown.

**Methods:**

To evaluate the role of Abl in asthma pathology, we assessed the expression of Abl in airway tissues from the ovalbumin sensitized and challenged mouse model, and human asthmatic airway smooth muscle cells. In addition, we generated conditional knockout mice in which Abl expression in smooth muscle was disrupted, and then evaluated the effects of Abl conditional knockout on airway resistance, smooth muscle mass, cell proliferation, IL-13 and CCL2 in the mouse model of asthma. Furthermore, we determined the effects of the Abl pharmacological inhibitors imatinib and GNF-5 on these processes in the animal model of asthma.

**Results:**

The expression of Abl was upregulated in airway tissues of the animal model of asthma and in airway smooth muscle cells of patients with severe asthma. Conditional knockout of Abl attenuated airway resistance, smooth muscle mass and staining of proliferating cell nuclear antigen in the airway of mice sensitized and challenged with ovalbumin. Interestingly, conditional knockout of Abl did not affect the levels of IL-13 and CCL2 in bronchoalveolar lavage fluid of animals treated with ovalbumin. However, treatment with imatinib and GNF-5 inhibited the ovalbumin-induced increase in IL-13 and CCL2 as well as airway resistance and smooth muscle growth in animals.

**Conclusions:**

These results suggest that the altered expression of Abl in airway smooth muscle may play a critical role in the development of airway hyperresponsiveness and airway remodeling in asthma. Our findings support the concept that Abl may be a novel target for the development of new therapy to treat asthma.

## Introduction

Asthma is a chronic airway disease characterized by airway hyperresponsiveness (AHR) and airway remodeling, which lead to impaired respiratory air flow in patients with asthma. However, the underlying mechanisms for the pathological processes are not fully understood.

AHR is largely attributed to hyperreactivity of airway smooth muscle [1]. The contractile properties of human asthmatic airway smooth muscle cells are distinctive from normal human airway smooth muscle cells [2,3]. In addition, the hyperreactivity of airway smooth muscle tissues occurs in animal models of asthma [4,5]. Furthermore, increased airway smooth muscle cell proliferation contributes to the progression of airway remodeling in asthma [6,7]. Airway smooth muscle hyperplasia may facilitate the thickening of the bronchial wall and promote AHR to a variety of stimuli [7,8].

Abl (Abelson tyrosine kinase, c-Abl) is a non-receptor tyrosine kinase that participates in the regulation of a range of cellular functions including migration and adhesion of nonmuscle cells [9,10]. Recent studies have implicated Abl in the regulation of vascular smooth muscle contraction *in vitro*. Contractile activation induces Abl phosphorylation, an indication of Abl activation [10,11], in smooth muscle. Knockdown of Abl attenuates smooth muscle force development in response to contractile activation [11-13]. Moreover, Abl has been implicated in regulating smooth muscle cell proliferation. Activation of Abl occurs in smooth muscle cells in response to stimulation with growth factors. Silencing of Abl inhibits smooth muscle cell proliferation induced by growth factors [14,15]. Nevertheless, the role of Abl in asthma pathology *in vivo* is largely unknown.

In this study, we generated smooth muscle-specific conditional knockout mice, and determined whether smooth muscle-specific knockout of Abl affects AHR and airway remodeling in a mouse model of chronic asthma. Our results suggest that the altered expression of Abl in airway smooth muscle is critical for the development of AHR and airway remodeling.

## Methods

### Animals and measurement of airway resistance

**Figure 1:**
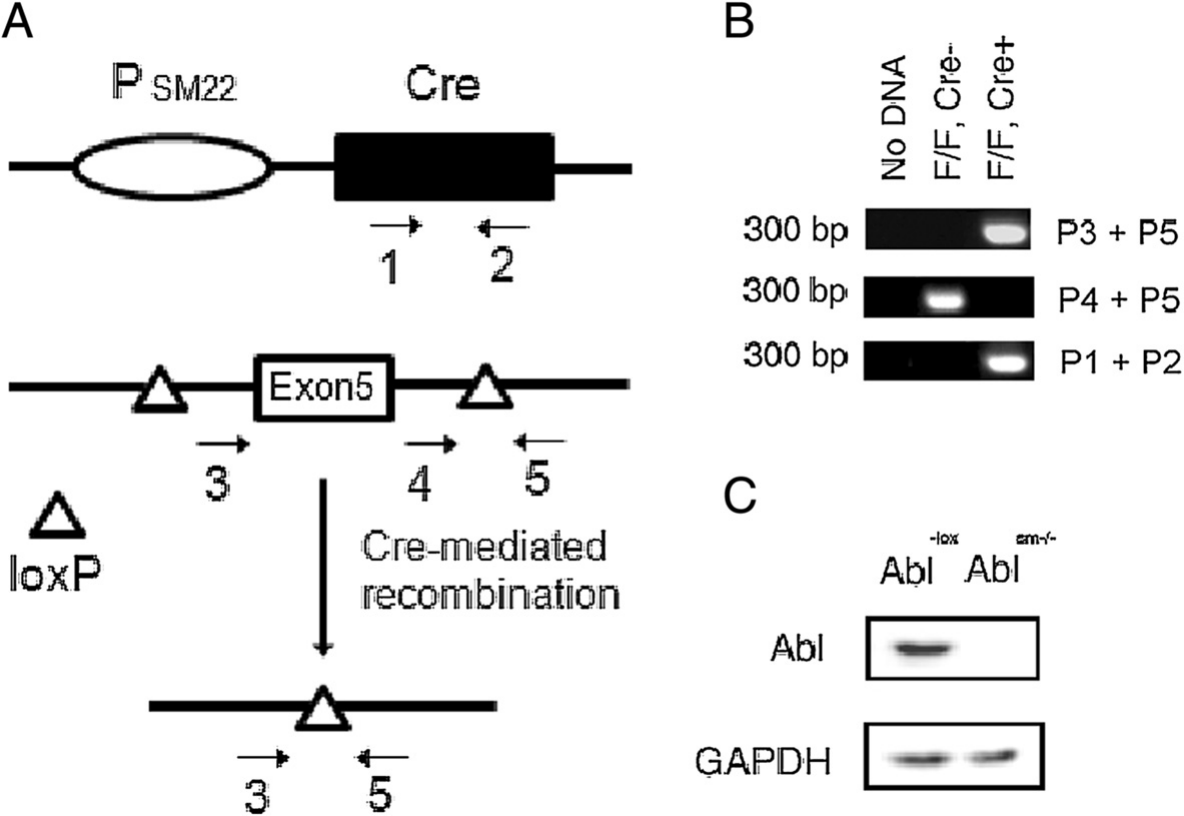
**Targeting of the *abl *gene in smooth muscle cells. (A)** SM22-Cre construct possesses a SM22 promoter (P_SM22_) and a Cre coding region. The *abl* locus containing a floxed version of the Exon5 of *abl* and the predicted product of Cre-mediated recombination are also shown. Numbered arrows represent PCR primers. **(B)** Ethidium bromide-stained agarose gel of PCR products amplified from mouse tails of indicated mouse strains. PCR with primers 3 and 5 demonstrate selective deletion of DNA between loxP sites in the presence of Cre recombinase in vivo. Each lane represents tail DNA samples isolated from an individual mouse of the indicated genotype. **(C)** Extracts of airway smooth muscle cells from Abl^-lox^ and Abl^sm−/−^ mice were analyzed by immunoblotting. Knockout of the Abl protein is verified in airway smooth muscle. Representative blots from three separate experiments are shown.

Abl^-lox^ mice were a gift of Dr. Koleske of Yale University [16]. SM22^cre^ mice were purchased from The Jackson Laboratory. Abl^-lox^ mice (genetic background, 129/Svj) were crossed with SM22^cre^ mice on C57BL/6 background. These mice express Cre recombinase under control of a smooth muscle-specific SM22 promoter. As a consequence, this loxP flanked exon 5 of the *abl* gene was excised in smooth muscle cells (Figure 1A). The sequences of primers used to identify the genotype were: Primer 1, 5’-CTGTACGTGTCCTCCGAGAG-3’; Primer 2, 5’-CTTCAAGGTCTTCACGGCCA-3’; Primer 3, 5’-GATGTCTCTACAGGGTTAAGATTAAGAGCA-3’; Primer 4, 5’-TGTGCATAGCAGGAAGTCCTCCAGAG-3’; Primer 5, 5’-AGTTAACACACCTCCAGAGTGAGTGCCCT-3’.

**Figure 2:**
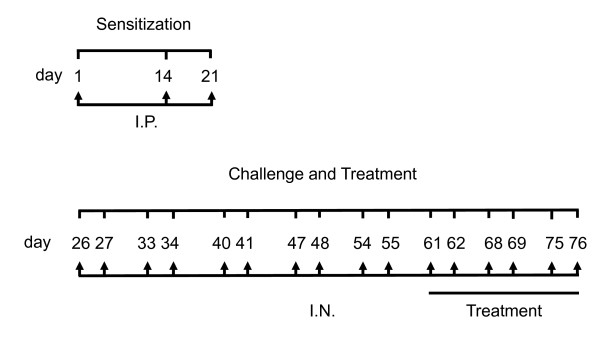
**Sensitization, challenge and treatment protocol for the animal model of chronic asthma.** I.P., intraperitoneal injection. I.N., intranasal instillation.

Age- and sex-matched Abl^-lox^ (control) mice and Abl^sm−/−^ (smooth muscle specific knockout of *abl* gene) mice (6–7 weeks old) were sensitized by intraperitoneal injection of sterile LPS-free ovalbumin (OVA) (Pierce) or phosphate buffered saline (PBS, control) for three weeks, and challenged with intranasal instillations of OVA or PBS twice a week for eight weeks using pre-viously-described protocols [17] with minor modification (Figure 2). To measure airway resistance, mice were anesthetized with an intraperitoneal injection of pentobarbital sodium, tracheotomized, and connected to the FlexiVent system (SCIREQ, Montreal, Canada) on Day 77. Mice were mechanically ventilated at 150 breaths/minute with a tidal volume of 10 ml/kg and a positive end-expiratory pressure (PEEP) of 3.35 cm H_2_O. Following baseline measurements, mice were challenged with methacholine (MCh) aerosol for 10 seconds at different doses. Airway resistance was measured for each mouse after inhalation of the aerosol. Dose–response curve was then determined. This study was approved by the Institutional Committee of Animal Care and Usage of Albany Medical College.

To assess the effects of inhibitors, BALB/c mice (6–7 weeks old) were purchased from The Jackson Laboratory. They were sensitized and challenged by OVA as described above. In addition, animals were intranasally instilled with 10 mg/kg GNF-5 or imatinib, or PBS 1 h before OVA instillation and 5 h after OVA instillation for last three weeks (Figure 2). Airway resistance in these mice was then assessed on Day 77.

### Assessment of tracheal ring contraction

Mice were euthanized by injection of pentobarbital (140 mg/kg). A segment of tracheas (4–5 mm in length) was immediately removed and placed in physiological saline solution (PSS) containing 110 mM NaCl, 3.4 mM KCl, 2.4 mM CaCl_2_, 0.8 mM MgSO_4_, 25.8 mM NaHCO_3_, 1.2 mM KH_2_PO_4_, and 5.6 mM glucose. The solution was aerated with 95%O_2_-5%CO_2_ to maintain a pH of 7.4. Two stainless steel wires were passed through the lumen of tracheal rings. One of the wires was connected to the bottom of organ baths and the other was attached to a Grass force transducer that had been connected to a computer with A/D converter (Grass). Tracheal segments were then placed in PSS at 37°C. Passive tension with 0.5 g was applied to each segment for 60 min. Contractile force in response to various treatments was then measured.

### Cell culture

Human airway smooth muscle (HASM) cells were prepared from human bronchi and adjacent tracheas obtained from the International Institute for Advanced Medicine [15]. Human tissues were non-transplantable and consented for research. This study was approved by the Albany Medical College Committee on Research Involving Human Subjects. Briefly, muscle tissues were incubated for 20 min with dissociation solution [130 mM NaCl, 5 mM KCl, 1.0 mM CaCl_2_, 1.0 mM MgCl_2_, 10 mM Hepes, 0.25 mM EDTA, 10 mM D-glucose, 10 mM taurine, pH 7, 4.5 mg collagenase (type I), 10 mg papain (type IV), 1 mg/ml BSA and 1 mM dithiothreitol]. All enzymes were purchased from Sigma-Aldrich. The tissues were then washed with Hepes-buffered saline solution (composition in mM: 10 Hepes, 130 NaCl, 5 KCl, 10 glucose, 1 CaCl_2_, 1 MgCl_2_, 0.25 EDTA, 10 taurine, pH 7). The cell suspension was mixed with Ham’s F12 medium supplemented with 10% (v/v) fetal bovine serum (FBS) and antibiotics (100 units/ml penicillin, 100 μg/ml streptomycin). Cells were cultured at 37°C in the presence of 5% CO_2_ in the same medium. The medium was changed every 3–4 days until cells reached confluence, and confluent cells were passaged with trypsin/EDTA solution [15,18,19]. Smooth muscle cells within passage 5 were used for the studies.

### Immunoblot analysis

Cells were lysed in SDS sample buffer composed of 1.5% dithiothreitol, 2% SDS, 80 mM Tris–HCl (pH 6.8), 10% glycerol and 0.01% bromophenol blue. The lysates were boiled in the buffer for 5 min and separated by SDS-PAGE. Proteins were transferred to a nitrocellulose membrane. The membrane was blocked with bovine serum albumin or milk for 1 h and probed with use of primary antibody followed by horseradish peroxidase-conjugated secondary antibody (Fisher Scientific). Proteins were visualized by enhanced chemiluminescence (Fisher Scientific) using the LAS-4000 Fuji Image System. Abl antibody was purchased from BD Biosciences and Santa Cruz Biotechnology. Glyceraldehyde 3-phosphate dehydrogenase (GAPDH) antibody was purchased from Fitzgerald (Acton, MA). The levels of proteins were quantified by scanning densitometry of immunoblots (Fuji Multigauge Software). The luminescent signals from all immunoblots were within the linear range.

### Immunohistochemistry

Mouse lungs were placed in frozen tissue-embedding medium (Neg 52, Richard-Allen Scientific) and cryosectioned using Cryostats (Richard-Allen Scientific). Tissue sections were fixed for 15 min in 4% paraformaldehyde, and were then washed three times in PBS buffer followed by permeabilization with 0.2% Triton X-100 dissolved in PBS for 5 min. These tissues were incubated with α-smooth muscle actin antibody (Sigma) or proliferating cell nuclear antigen (PCNA) antibody (Thermo Scientific) followed by appropriate secondary antibody conjugated to Alexa-488 or Alex-543 (Molecular Probes/Life Technologies). The sections were also counterstained with 4',6-diamidino-2-phenylindole to visualize the nucleus. The samples were viewed and digitally captured using a Leica microscope system (MDI 6000). All immunohistochemical measurements were performed by using the NIH ImageJ software.

### Analysis of airway inflammation

Lungs from sacrificed mice were lavaged three times with 1 mL sterile Hanks balanced salt solution (HBSS) containing 3 mM EDTA. Bronchoalveolar lavage fluid (BALF) was collected after centrifugation and, the supernatant was removed and frozen at −80°C for cytokine/chemokine measurements (*See below*). The cell pellet was resuspended in HBSS, and total number of inflammatory cells in the BALF was counted by using a hemocytometer. Differential cell counts (macrophages, neutrophils, lymphocytes, and eosinophils) were performed by counting 100 cells from cytospin preparations stained with DiffQuick stain. The levels of IL-13 and CCL2 in the BALF were determined using ELISA kits (R&D systems) according to the manufacturer’s instructions.

### Statistical analysis

All statistical analysis was performed using Prism 4 software (GraphPad Software, San Diego, CA). Comparison among multiple groups was performed by one-way analysis of variance followed by Tukey’s multiple comparison test. Differences between two groups were analyzed by Student-Newman-Keuls test or Dunn's method. Values of n refer to the number of experiments used to obtain each value. P < 0.05 was considered to be significant.

## Results

### Abl is required for airway smooth muscle contraction

Our previous studies demonstrate that Abl regulates vascular smooth muscle contraction [11-13]. To determine the role of Abl in airway smooth muscle, we generated SM22^cre^Abl^-lox^ mice, a mouse model with smooth muscle cell–specific disruption of the *abl* gene (Figure 1A). Genotyping and immunoblot analysis verified knockout of Abl in airway smooth muscle (Figure 1B and C). Previous studies by others demonstrate that SM22 is expressed in airway smooth muscle tissues [20-22], which suggests that SM22 promoter is functional in airway smooth muscle. Our results are consistent with these studies.

**Figure 3:**
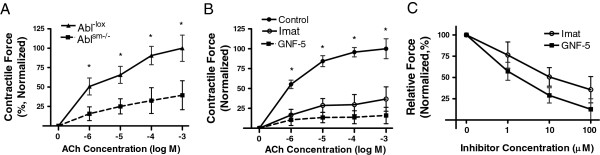
**Abl is required for airway smooth muscle contraction. (A)** Tracheal rings of Abl^sm−/−^ mice and Abl^-lox^ (control) mice were treated with difference concentration of acetylcholine (ACh). Dose–response of these rings was then evaluated. Contractile force is normalized to maximal force of tracheal rings from Abl^-lox^ mice. Values are mean ± SE (n = 9–10). * Significantly lower active force in Abl^sm−/−^ mice than in Abl^-lox^ at corresponding doses (P < 0.01). **(B)** Tracheal rings of Abl^-lox^ mice were pretreated with or without the Abl inhibitors imatinib or GNF-5 (10 μM, 10 min). ACh dose–response of tissues was then determined. Pretreatment with imatinib or GNF-5 attenuated ACh-induced contraction *in vitro* (n = 15–18, * P < 0.01). **(C)** Tracheal rings of Abl^-lox^ mice were precontracted with 100 μM ACh. Different concentrations of imatinib or GNF-5 were then imposed to assess the relaxation response. Treatment with the pharmacological inhibitors induced relaxation of tracheal segments precontracted by ACh (n = 17–19).

Contractile responses of mouse tracheal rings to ACh stimulation were compared between Abl^sm−/−^ (smooth muscle knockout of Abl) mice and Abl^-lox^ (control) mice. As shown in Figure 3A, contractile responses of mouse tracheal rings to ACh were lower in Abl^sm−/−^ mice than in Abl^-lox^ mice, which was dose-dependent.

We also evaluated acute effects of the Abl pharmacological inhibitors imatinib (Gleevec, STI-571) [11,23] and GNF-5 [24] on airway smooth muscle contraction. Treatment of mouse tracheal rings with imatinib significantly attenuated force development induced by ACh (Figure 3B). Likewise, GNF-5 had inhibitory effects on contraction of tracheal segments with slightly stronger potency (Figure 3B). Furthermore, treatment with imatinib or GNF-5 induced relaxation of tracheal rings precontracted by ACh (Figure 3C).

### The expression of Abl is upregulated in asthmatic airway smooth muscle

**Figure 4:**
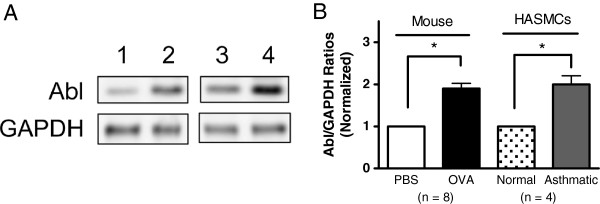
**The expression of Abl is higher in asthmatic airways tissues**/**cells. (A)** The upregulation of Abl expression in the airways of ovalbumin (OVA)-sensitized and challenged mice and human airway smooth muscle (HASM) cells of patients with severe asthma. Blots of tissue/cell extracts were probed with use of antibodies against Abl and GAPDH. 1, tracheal extracts of naïve mice; 2, tracheal extracts of OVA-sensitized and challenged mice; 3, normal HASM cell extracts; 4, asthmatic HASM cell extracts. **(B)** For quantification analysis, Abl/GAPDH ratios in OVA-treated animals or asthmatic HASM cells are normalized to corresponding control tissues or cells (*P < 0.05).

We evaluated the expression of Abl in airway tissues of OVA-sensitized and challenged mice, a well-recognized animal model mimicking allergen-induced asthma in humans [17]. As shown in Figure 4A, the amount of Abl was elevated in airway tissues of OVA-treated mice compared to naïve animals. However, the levels of GAPDH were similar in OVA-treated mice and naïve mice. The ratio of Abl/GAPDH in airway tissues was higher in OVA-treated mice than in naïve mice (Figure 4B). To validate this finding in human asthma, we assessed Abl expression in HASM cells from normal subjects and patients with severe asthma. The level of Abl was higher in asthmatic cells than in normal cells. The ratios of Abl/GAPDH in asthmatic cells were significantly higher as compared to normal cells (Figure 4A and B).

### Conditional knockout of Abl in smooth muscle attenuates OVA-sensitized airway resistance and contraction of tracheal rings

**Figure 5:**
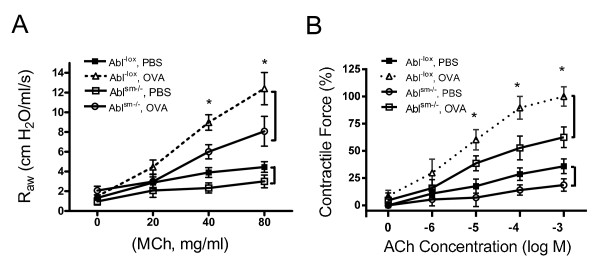
**Abl knockout attenuates airway resistance and contraction of tracheal rings in the animal model of asthma. (A)** Abl^-lox^ and Abl^sm−/−^ mice were sensitized and challenged by OVA. Airway resistance (R_AW_) in these mice was evaluated using the FlexiVent system. R_AW_ in Abl^-lox^ mice treated with OVA was higher than in Abl^-lox^ mice treated with PBS, verifying the development of AHR in the mouse model. R_AW_ in Abl^sm−/−^ mice treated with OVA was reduced compared to Abl^-lox^ mice treated with OVA (n = 10–14, *P < 0.05). **(B)** The contractile response of tracheal rings from these mice was determined by using an *in vitro* organ bath system. Contractile force is normalized to maximal force of rings from OVA-treated Abl^-lox^ mice. Contractile force of OVA-sensitized and challenged Abl^-lox^ mice was increased as compared to naïve Abl^-lox^ mice. OVA-induced airway smooth muscle contraction was reduced in Abl^sm−/−^ mice (n = 10–14, *P < 0.05).

We used a chronic asthma animal model [17] to determine whether Abl in smooth muscle is involved in AHR. Briefly, Abl^-lox^ and Abl^sm−/−^ mice were sensitized by OVA for three weeks and challenged by OVA for eight weeks (Figure 2). Airway resistance in response to methacholine (MCh) inhalation was measured using the FlexiVent system. OVA sensitization and challenge induced a higher response to MCh inhalation in Abl^-lox^ mice as compared to Abl^-lox^ mice treated with PBS. However, airway resistance induced by MCh was lower in Abl^sm−/−^ mice sensitized and challenged by OVA than in Abl^-lox^ mice treated with OVA. The airway resistance was also lower in naïve Abl^sm−/−^ mice than in naïve Abl^-lox^ mice (Figure 5A).

We also assessed the effects of Abl knockout on airway smooth muscle hyperreactivity *in vitro*. Contractile force in isolated tracheal rings from OVA-treated Abl^-lox^ mice was greater compared to naïve Abl^-lox^ mice. However, active force of isolated tracheal rings from OVA-treated Abl^sm−/−^ mice was reduced compared to OVA-treated Abl^-lox^ mice. Contractile response of tracheal rings from naïve Abl^sm−/−^ mice was also lower compared to naïve Abl^-lox^ mice (Figure 5B).

### Pharmacological inhibition of Abl diminishes AHR and smooth muscle hyperreactivity

**Figure 6:**
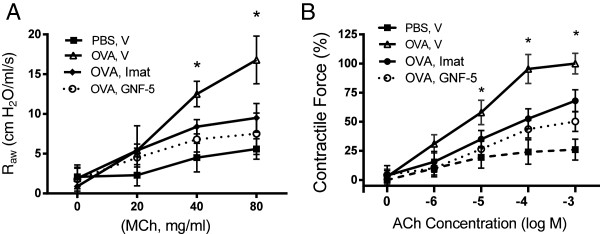
**Effects of imatinib and GNF**-**5 on airway resistance and contractile response of tracheal rings from OVA**-**sensitized and challenged mice. (A)** BALB/c mice were sensitized and challenged with OVA in the presence of imatinib, GNF-5 or vehicle (V). Airway resistance in these mice was then measured. Intranasal instillation of imatinib and GNF-5 inhibited R_AW_ in mice sensitized and challenged by OVA. *Significantly lower airway resistance in animals treated with imatinib or GNF-5 compared to animals treated with vehicle (P < 0.05, n = 7–8). **(B)** Treatment with imatinib and GNF-5 attenuated the OVA-sensitized tracheal contraction *in vitro*. Contractile force is normalized to maximal force of rings from OVA- and vehicle-treated mice. *Significantly lower tracheal contraction from animals treated with imatinib or GNF-5 compared to vehicle-treated animals (P < 0.05, n = 7–8).

We also evaluated the effects of the Abl inhibitors imatinib and GNF-5 on AHR in asthmatic animals. The OVA sensitization and challenge increased airway resistance in BALB/c mice as compared to BALB/c mice treated with PBS (Figure 6A). In contrast, the OVA-induced increase in airway resistance was reduced in the animals treated with imatinib or GNF-5 (Figure 6A). In addition, treatment with imatinib or GNF-5 inhibited the ACh-induced contraction in isolated mouse tracheal rings of OVA-sensitized and challenged mice (Figure 6B).

We noticed that airway resistance in response to MCh inhalation was slightly higher in BALB/c mice than in Abl^-lox^ mice sensitized and challenged by OVA (Figure 5A and Figure 6A). This is not surprising because BALB/c mouse strain is known to have skewed Th2 response compared to other mouse strains [25,26].

### Conditional knockout of Abl inhibits airway smooth muscle growth in the animal model of asthma

To determine the role of Abl in the remodeling of airway smooth muscle, we assessed whether conditional knockout of Abl in smooth muscle affects the allergen-induced airway smooth muscle mass by determining the area of α-smooth muscle actin (a smooth muscle marker) staining in the airways of Abl^-lox^ and Abl^sm−/−^ mice sensitized and challenged with OVA.

**Figure 7:**
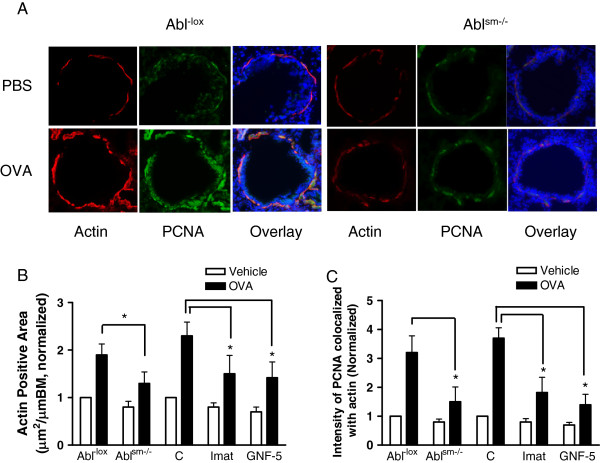
**Abl knockout inhibits airway remodeling in the animal model of asthma. (A)** Representative images illustrating the effects of Abl knockout on α-actin and PCNA staining. Abl^-lox^ and Abl^sm−/−^ mice were sensitized and challenged with OVA. Lung sections of the mice were immunostained for α-actin (red) and PCNA (green). The sections were also counterstained with use of 4',6-diamidino-2-phenylindole to visualize the nucleus (blue). **(B)** Actin positive area in Abl^sm−/−^ mice treated with OVA was lower compared to Abl^-lox^ mice exposed to OVA (n = 6–7, *P < 0.05). Treatment with imatinib or GNF-5 inhibited the OVA-induced increase in actin positive area from BALB/c mice (n = 6–7, *P < 0.05). C, control animals. **(C)** Fluorescent intensity of PCNA colocalized with actin in the airways in Abl^sm−/−^ mice treated with OVA was lower than that in Abl^-lox^ mice exposed to OVA (n= 6–7, *P < 0.05). Treatment with imatinib or GNF-5 inhibited the OVA-induced increase in PCNA staining from BALB/c mice (n = 6–7, *P < 0.05). C, control animals.

The area of α-smooth muscle actin staining in the airways of Abl^-lox^ mice treated with OVA was greater than that in Abl^-lox^ mice treated with PBS, as evidenced by immunofluorescent analysis. In contrast, the area of actin staining in the airways of Abl^sm−/−^ mice treated with OVA was reduced as compared to Abl^-lox^ mice treated with OVA (Figure 7A & B). These results suggest that conditional knockout of Abl is able to attenuate the allergen-induced increase in airway smooth muscle mass. In addition, the fluorescent intensity of α-smooth muscle actin staining was higher in Abl^-lox^ mice treated with OVA compared to naïve Abl^-lox^ mice, suggesting higher α-smooth muscle actin expression in the remodeled airway in asthmatic models [6]. Furthermore, we determined the effects of imatinib or GNF-5 on airway smooth muscle growth. Treatment with the inhibitors attenuated an increase in airway smooth muscle mass in BALB/c mice sensitized and challenged by OVA (Figure 7B).

We also evaluated whether the knockout of Abl influences allergen-induced airway smooth muscle cell proliferation. Proliferating cell nuclear antigen (PCNA) is a critical protein that is expressed by proliferating cells in S phase of the cell cycle. Thus, it has been widely used as a marker of cell proliferation in the airways [8,27]. The fluorescent intensity of PCNA colocalized with α-smooth muscle actin was greater in the airways of Abl^-lox^ mice treated with OVA compared with Abl^-lox^ mice treated with PBS. However, the intensity of PCNA costained with actin in the airways of Abl^sm−/−^ mice treated with OVA was lower than that in the airways of Abl^-lox^ mice treated with OVA (Figure 7A and C). Moreover, treatment with the Abl inhibitors imatinib or GNF-5 diminished the fluorescent intensity of PCNA in BALB/c mice treated with OVA (Figure 7C). These results indicate that Abl has a role in the allergen-induced airway smooth muscle proliferation.

### Effects of conditional knockout of Abl and Abl inhibitors on airway inflammation in the animal model of asthma

As a consequence of allergic sensitization and challenge, inflammatory cells enter into the lungs and cytokine/chemokine levels are increased in the bronchoalveolar space of asthmatic patients and animal models, which are characteristic features of allergic airway inflammation [7,28,29]. To determine whether the smooth muscle-specific depletion of Abl affects recruitment of inflammatory cells, we determined total and differential cell counts of BALF in lungs of naïve and OVA-treated Abl^-lox^ mice and Abl^sm−/−^ mice.

**Figure 8:**
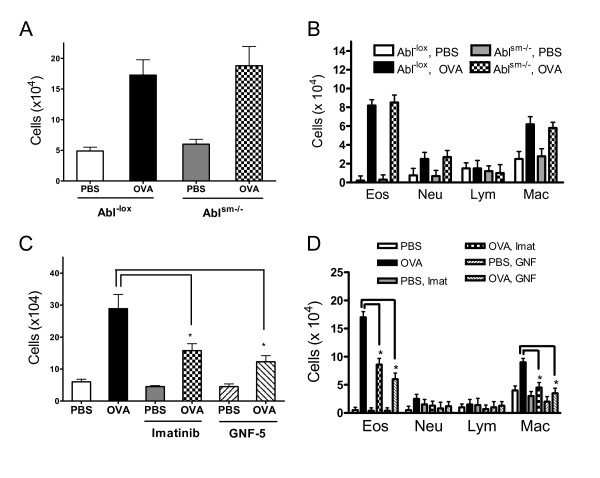
**Effects of Abl knockout**, **imatinib and GNF**-**5 on total and differential cell counts in BALF.** Total **(A)** and differential **(B)** cell numbers in BALF of Abl^sm−/−^ mice were similar to those of Abl^-lox^ mice when treated with PBS or OVA (n = 6–7). However, treatment with imatinib or GNF-5 inhibited the OVA-induced increase in total **(C)** and differential **(D)** cell numbers in BALF of BALB/c mice (n = 7, *P < 0.05).

OVA sensitization and challenge increased the numbers of total and differential cells in the lungs of Abl^-lox^ mice. However, the allergen-induced increase in cell numbers in the lungs in Abl^sm−/−^ mice was similar to that in Abl^-lox^ mice (Figure 8A and B).

We also evaluated the effects of the Abl inhibitors imatinib and GNF-5 on cell counts of BALF from mice treated with PBS or OVA. OVA sensitization and challenge increased total and differential cell counts of BALF from BALB/c mice. Treatment with imatinib and GNF-5 reduced the OVA-induced increase in inflammatory cell numbers (Figure 8C and D).

**Figure 9:**
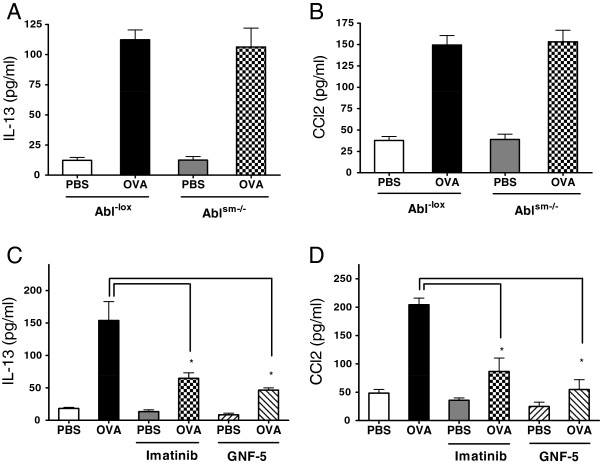
**Effects of Abl knockout**, **imatinib and GNF**-**5 on the levels of IL**-**13 and CCL2 in BALF. (A)** The level of IL-13 in BALF of Abl^sm−/−^ mice was similar to those of Abl^-lox^ mice treated with PBS or OVA (n = 7). **(B)** Abl conditional knockout did not affect the concentration of CCL2 in BALF (n = 6). **(C)** The OVA-induced increase in IL-13 level in BALF from BALB/c mice was attenuated by treatment with imatinib or GNF-5 (n= 7, *P < 0.05). **(D)** Treatment with imatinib or GNF-5 inhibited the OVA-induced enhancement of CCL2 in BALF from BALB/c mice (n = 6, *P < 0.05).

To determine the role of Abl in smooth muscle in the production of cytokine and chemokine, we evaluated the level of IL-13 and CCL2 (representative cytokine and chemokine in asthma pathology) [5,30,31] in the BALF in lungs of naïve and OVA-treated Abl^-lox^ and Abl^sm−/−^ mice. OVA sensitization and challenge increased the level of IL-13 and CCL2 in the BALF of Abl^-lox^ mice. In addition, the allergen-induced increase in IL-13 and CCL2 in the lungs of Abl^sm−/−^ mice was similar to those in Abl^-lox^ mice (Figure 9A and B). However, treatment with imatinib and GNF-5 diminished the OVA-induced increase in IL-13 and CCL2 in the lungs of BALB/c mice (Figure 9C and D).

## Discussion

Abl is a non-receptor tyrosine kinase that has a role in regulating smooth muscle contraction and smooth muscle cell proliferation *in vitro*. The role of Abl in the pathogenesis of asthma *in vivo* is largely unknown. In this study, Abl expression is upregulated in asthmatic airways. More importantly, conditional knockout of Abl in smooth muscle inhibits airway resistance and airway smooth muscle growth in the animal model of chronic asthma. The results suggest that Abl plays a critical role in the progression of AHR and airway remodeling in chronic asthma.

Our previous studies demonstrate that Abl is essential for vascular smooth muscle force development [10-13]. In this report, conditional knockout of Abl in smooth muscle diminished contractile response of tracheal rings. Moreover, acute inhibition of Abl by the pharmacological agents attenuated contraction in tracheal rings. The results suggest that Abl is necessary for airway smooth muscle contraction. Abl may regulate the functional states of several proteins including Crk-associated substrate and Abi1, which in turn regulate actin dynamics and smooth muscle contraction [11-13,32,33].

AHR largely stems from hyperreactivity of airway smooth muscle [1]. The pathological mechanisms that mediate airway smooth muscle hyperreactivity and AHR in asthma are not completely elucidated. Th2 cytokines including IL-13 has been implicated in smooth muscle hypercontractility and AHR [34-36]. In this study, the expression of Abl was upregulated in airway tissues of the animal model of asthma as well as in smooth muscle cells of patients with severe asthma. Furthermore, conditional knockout of Abl in smooth muscle attenuated airway smooth muscle hyperreactivity *in vitro* and airway resistance in mice sensitized and challenged by the allergen. To rule out the potential effects by compensation in genetically-modified mice, we also determined the acute effects of the Abl pharmacological inhibitors imatinib [11,23] and GNF-5 [24] on airway resistance *in vivo* and airway smooth muscle hyperreactivity *in vitro*. Treatment with the inhibitors also diminished the OVA-sensitized airway resistance *in vivo* and tracheal contraction *in vitro*. The results suggest that Abl has a critical role in the development of AHR in asthma.

Airway remodeling is a characteristic feature of severe asthma. In addition to fibrosis, enhanced deposition of extracellular matrix protein, epithelial injury and airway smooth muscle hypertrophy, proliferation of airway smooth muscle cells markedly contributes to the pathogenesis of airway remodeling [6,8,29,37]. Our recent studies demonstrate that Abl is required for smooth muscle cell proliferation in *in vitro* studies [14,15]. Abl may modulate cell proliferation by affecting actin polymerization and the Raf-1/MEK/ERK1/2 pathway [14,15].

Growth factors such as epidermal growth factor and platelet-derived growth factor have been implicated in the progression of airway remodeling [7]. In this report, smooth muscle mass in the airways was reduced in conditional knockout mice sensitized and challenged by ovalbumin. In addition, the cell proliferation marker PCNA was also diminished in conditional knockout mice treated with the allergen. Moreover, treatment with the pharmacological inhibitors had similar effects. Thus, the increased expression of Abl in smooth muscle may contribute to the development of airway remodeling in chronic asthma.

In response to allergic sensitization and challenge, inflammatory cells enter into the lungs and cytokine/chemokine levels are increased in the bronchoalveolar space of asthmatic patients and animal models [4,30,34]. Because airway smooth muscle cells have ability to secret cytokines *in vitro*[28], we assessed whether Abl knockout in smooth muscle affects airway inflammation. Conditional knockout of Abl did not affect the increase in inflammatory cell numbers, IL-13 and CCL2 in animals sensitized and challenged by the allergen. The results lead us to suggest that Abl expression in smooth muscle does not modulate inflammatory cell infiltration and production of IL-13 and CCL2 in asthma.

On the contrary, treatment with imatinib and GNF-5 reduced the OVA-induced increase in inflammatory cell numbers, and levels of IL-13 and CCL2. The results suggest that global inhibition of Abl diminishes airway inflammation in chronic asthma, which is consistent with the findings that Abl may regulate migration and synthetic functions of immune cells *in vitro*[38-41].

Currently, β_2_ agonists are widely used to treat asthma. β_2_ agonists reduce symptoms of airway obstruction by inducing airway smooth muscle relaxation. However, this therapy has various limitations including β_2_ adrenergic receptor desensitization [42]. In this study, we demonstrate that Abl in smooth muscle has a critical role in the pathogenesis of AHR and airway remodeling. Furthermore, global inhibition of Abl by pharmacological agents attenuates airway inflammation. Thus, our findings support the concept that Abl may be a novel target for the development of new therapy to treat asthma.

## Conclusions

Abl is a non-receptor tyrosine kinase that has a role in regulating smooth muscle contraction and smooth muscle cell proliferation *in vitro*. The role of Abl in asthma pathogenesis *in vivo* is not well elucidated. Our present results suggest that the altered expression of Abl in smooth muscle plays a critical role in the progression of AHR and airway remodeling in chronic asthma. Furthermore, global inhibition of Abl attenuates airway inflammation. Thus, Abl may be a novel target for the development of new therapy to treat asthma.

## Abbreviations

Abl: Abelson tyrosine kinase; ACh: acetylcholine; AHR: airway hyperresponsiveness; BALF: Bronchoaleolar lavage fluid; CCL2: Chemokine (C-C motif) ligand 2; GAPDH: Glyceraldehyde 3-phosphate dehydrogenase; GNF-5: bcr-Abl inhibitor III; HASM: Human airway smooth muscle; IL-13: Interleukin-13; MCh: Methacholine; OVA: Ovalbumin.

## Competing interests

The authors declare that they have no competing interests.

## Authors’ contributions

RAC performed animal studies. RW carried out cell culture studies and participated in animal studies and immunohistochemical studies. TW performed *in vitro* contraction studies. DDT designed the experiments, analyzed data and wrote the manuscript. All authors read and approved the final manuscript.
